# Pneumatocele formation in adult *Escherichia coli* pneumonia

**DOI:** 10.4103/1817-1737.78434

**Published:** 2011

**Authors:** M.M. Puri, A. Srivastava, A.K. Jain, D. Behera

**Affiliations:** *Department of Tuberculosis and Respiratory Diseases, LRS Institute of Tuberculosis and Respiratory Diseases, New Delhi, India. Email: mmpuri@rediffmail.com*

Sir,

Pulmonary pneumatoceles are acquired, thin-walled, air-containing transient lesions of the lungs, commonly seen as a sequela of staphylococcal pneumonia in infants and young children.[1–3] They are infrequent in adults and the offending organism is usually *Staphylococcus aureas* or *Pneumococcus*. We report of a case of *Escherichia coli* (*E. coli*) pneumonia with pneumatocele formation. A 42–year-old female, known case of bronchial asthma on therapy with inhaled corticosteroids and long acting β-2 agonist for 4 years developed fever, cough, expectoration, breathlessness, chest pain, anorexia, weight loss, and diarrhea for 3 weeks. She was also taking oral corticosteroids (20-30 mg prednisolone per day) for the last 6 months. She was admitted in an emergency ward in respiratory distress with a respiratory rate of 24/min, pulse rate 117/ min, blood pressure 110/80 mmHg, body temperature 100°F, oxygen saturation (PSO_2_) 93% at oxygen flow of 4 l/min. Physical examination revealed intercostal retraction and use of sternocleidomastoid and trapezius muscle, coarse crackles, and rhonchi bilaterally. A chest radiograph revealed multiple rounded homogenous parenchymal shadows of varying size, 2-5 cm in diameter in both lung fields [Figure 1a]. Her blood hemoglobin was 12.0 g%, total leucocytes counts were 22,000/cu. mm with 70% polymorphs and 30% lymphocytes. Arterial blood examination revealed pH = 7.55, PaCO_2_ = 45.7 mmHg, PaO_2_= 61.7 mmHg, HCO–_3_ = 40.3 mmol/l while breathing oxygen at the rate of 3 l/ min by a nasal cannula. She was treated with nebulised salbutamol and ipratropium bromide, intravenous hydrocortisone, ceftriaxone 1 g intravenously 12 hourly, and gentamycin 120 mg intravenously daily. Sputum culture grew *E. coli* sensitive to gentamycin and amikacin and resistant to cephotaxime, ceftazidime, ciprofloxacin, ceftriaxone, and tobramycin. Blood culture was sterile after 72 h of incubation. Injection ceftriaxone was stopped and Injection gentamycin 120 mg intravenously daily was continued along with capsule Clindamycin 600 mg thrice a day and amoycillin–clavulanic acid 1.2 g intravenously eight hourly. After a week she improved and a chest radiograph revealed zones of radiolucency in the parenchymal shadows. After 2 weeks the patient again had high-grade fever and her sputum and urine culture grew *E. coli* sensitive to gentamycin and amikacin and resistant to cephotaxime, ceftazidime, ciprofloxacin, ceftriaxone, and tobramycin. A chest radiograph at this stage showed clearing of the opacities with thin-walled cavities [Figure 1b]. Her treatment with injection gentamycin 120 mg intravenously daily was continued for another 14 days. Treatment with combination of inhaled corticosteroids and long acting β-2 agonist was advised at discharge for bronchial asthma. Follow-up chest radiograph taken after 4 months showed regression of the pneumatoceles; however, few pneumatoceles were still persist on the chest computed tomography (CT) scan obtained at the same time

**Figure 1a:**
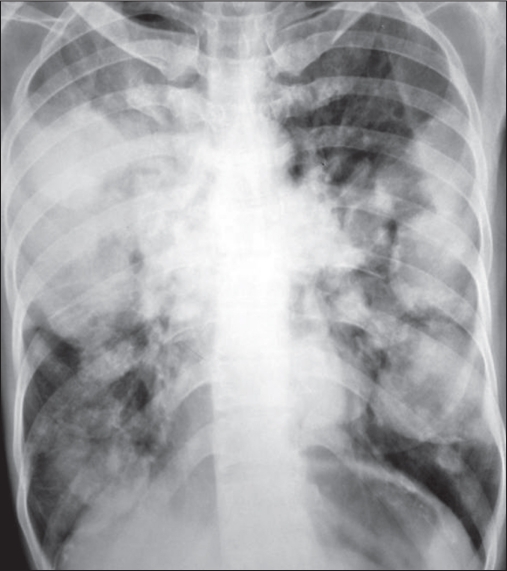
Chest X-ray PA view multiple rounded homogenous parenchymal shadows of varying size, 2–5 cm in diameter in both lung fields. Some of these shadows coalesce with each other and surrounding mediastinal structures

**Figure 1b:**
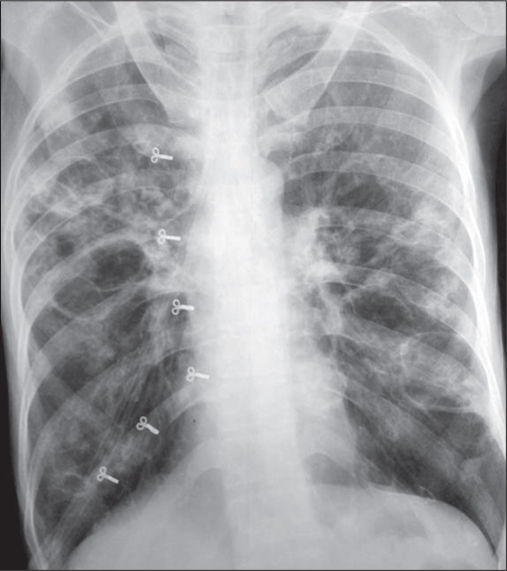
Chest X-ray PA view at 4 weeks showed clearing of the opacities with thin-walled cavities.

Pneumatoceles are acquired air cysts that develop after lung infection, pulmonary trauma (laceration), or hydrocarbon ingestion. They represent an area of localized pulmonary over inflation caused by transient bronchial or bronchiolar obstruction of the check-valve type.[4] The obstruction is believed to be caused by inflammatory exudates within the airway lumen or wall, allowing air to enter the cystic space but not to leave it. Pulmonary pneumatoceles are formed initially by drainage of necrotic lung parenchyma and subsequent enlargement of the pneumatocele caused by check-valve bronchiolar obstruction, which is due to either pressure from the adjacent pneumatocele or intraluminal inflammatory exudates. Pneumatoceles are generally observed soon after the development of pneumonia but can be observed on the initial chest radiograph. Radiographic evidence of a pneumatocele most often occurs on day 5–7 of hospitalization. They can be single but are more often multiple, thin-walled, air-filled, cyst-like cavities, or “ring shadows” greater than 1 cm in diameter with wall <4 mm and of uniform thickness.[5] No specific genetic factor is known to predispose individuals to pneumatocele formation. However, pneumatoceles are associated with hyperimmunoglobulinemia E syndrome (Buckley–Job syndrome), which predisposes the patient to staphylococcal pneumonia. Other organisms that may lead to this condition include *Streptococcus pneumoniae, Haemophilus influenzae, E. coli, S. pyogenes, Serratia marcescens, K. pneumoniae, adenovirus, M. tuberculosis, and Pneumocystis jiroveci*. Trauma and hydrocarbon ingestion may also play a role. *Pneumocysti carinii* is also a well-recognized cause of pneumatocele formation in adults. Even though *E. coli* pneumonia might be a common cause of pneumatocele in children, our Medline research has identified only one such case in adult patients.[6]

Patients on long-term corticosteroid therapy are at a risk of infections and complications. Colling *et al*. described pulmonary pneumatoceles complicating the early course of nosocomial *E. coli* pneumonia in a 51-year-old patient on long-term corticosteroid therapy for seronegative ankylosing spondylitis.[6] Their patient developed pneumothorax few days after digestive surgery and multiple pneumatoceles in the right lung were demonstrated after the removal of intercostal drainage tube in the chest radiograph and CT scan. Our patient was also on corticosteroid therapy for 6 months for control of bronchial asthma. She developed multiple pneumatoceles in both lungs during the course of recovery from pneumonia. *E. coli* pneumonia is almost always associated with E coli urinary tract infection (UTI). In our patient E. coli was also isolated in urine. *E. coli* pneumonia may also result from microaspiration of upper airway secretions that have been previously colonized with this organism in severely ill patients who have underlying diseases such as diabetes mellitus, alcoholism, and *E. coli* UTI.

In the majority of cases, pulmonary pneumatoceles resolve spontaneously within few weeks to months. However, rare complications can occur, including tension pneumatocele, pneumothorax, and secondarily infected pneumatocele. Medical care of the underlying condition is the first-line therapy. Invasive approaches should only be reserved for patients who develop complications. The clinical course of our patient improved gradually with antibiotic treatment.
